# Antimicrobial resistance in bacterial wound, skin, soft tissue and surgical site infections in Central, Eastern, Southern and Western Africa: A systematic review and meta-analysis

**DOI:** 10.1371/journal.pgph.0003077

**Published:** 2024-04-16

**Authors:** Edward J. M. Monk, Timothy P. W. Jones, Felix Bongomin, Winnie Kibone, Yakobo Nsubuga, Nelson Ssewante, Innocent Muleya, Lauryn Nsenga, V. Bhargavi Rao, Kevin van Zandvoort

**Affiliations:** 1 Department of Infectious Disease Epidemiology, London School of Hygiene and Tropical Medicine, London, United Kingdom; 2 Infection Care Group, St George’s University Hospitals NHS Foundation Trust, London, United Kingdom; 3 Nuffield Department of Medicine, Jenner Institute, University of Oxford, Oxford, United Kingdom; 4 Faculty of Medicine, Department of Medical Microbiology and Immunology, Gulu University, Gulu, Uganda; 5 Child and Health Development Centre, College of Health Sciences, Makerere University, Kampala, Uganda; 6 The Mason Unit, MSF UK, London, United Kingdom; 7 School of Medicine, Kabale University, Kabale, Uganda; 8 Department of Global Health and Development, London School of Hygiene and Tropical Medicine, London, United Kingdom; Kamuzu University of Health Sciences, MALAWI

## Abstract

Antimicrobial resistance (AMR) is a major global threat and AMR-attributable mortality is particularly high in Central, Eastern, Southern and Western Africa. The burden of clinically infected wounds, skin and soft tissue infections (SSTI) and surgical site infections (SSI) in these regions is substantial. This systematic review reports the extent of AMR from sampling of these infections in Africa, to guide treatment. It also highlights gaps in microbiological diagnostic capacity. PubMed, MEDLINE and Embase were searched for studies reporting the prevalence of *Staphylococcus aureus*, *Eschericheria coli*, *Klebsiella pneumoniae*, *Pseudomonas aeruginosa* and *Acinetobacter baumannii* in clinically infected wounds, SSTI and SSI in Central, Eastern, Southern or Western Africa, and studies reporting AMR from such clinical isolates. Estimates for proportions were pooled in meta-analyses, to estimate the isolation prevalence of each bacterial species and the proportion of resistance observed to each antibiotic class. The search (15^th^ August 2022) identified 601 articles: 59 studies met our inclusion criteria. *S*. *aureus* was isolated in 29% (95% confidence interval [CI] 25% to 34%) of samples, *E*. *coli* in 14% (CI 11% to 18%), *K*. *pneumoniae* in 11% (CI 8% to 13%), *P*. *aeruginosa* in 14% (CI 11% to 18%) and *A*. *baumannii* in 8% (CI 5% to 12%). AMR was high across all five species. *S*. *aureus* was resistant to methicillin (MRSA) in >40% of isolates. *E*. *coli* and *K*. *pneumoniae* were both resistant to amoxicillin-clavulanic acid in ≥80% of isolates and resistant to aminoglycosides in 51% and 38% of isolates respectively. *P*. *aeruginosa* and *A*. *baumannii* were both resistant to anti-pseudomonal carbapenems (imipenem or meropenem) in ≥20% of isolates. This systematic review found that a large proportion of the organisms isolated from infected wounds, SSTI and SSI in Africa displayed resistance patterns of World Health Organisation (WHO) priority pathogens for critical or urgent antimicrobial development.

## Introduction

Antimicrobial resistance (AMR) is recognised by the World Health Organization (WHO) as a major global health threat to humanity, estimated to lead to 10 million deaths annually by 2050 [1–3]. In a recent systematic review of the global burden of AMR on health, four sub-regions of Africa were all found to have rates of death attributable to bacterial AMR higher than any other global sub-region: Central, Eastern, Southern and Western Africa each had a AMR-attributable mortality rate of over 75/100,000 [4]. In their review, Murray et al found that six main pathogens contribute to AMR burden in these African sub-regions: *Staphylococcus aureus*, *Streptococcus pneumoniae*, *Escherichia coli*, *Klebsiella pneumoniae*, *Pseudomonas aeruginosa* and *Acinetobacter baumannii*. Of these, all but *S*. *pneumoniae* are recognised as common pathogens of wound infections, skin and soft tissue infections (SSTI) or surgical site infections (SSI) [5,6].

Globally, bacterial infections of the skin and subcutaneous tissues are the sixth highest infectious syndrome causing AMR-attributable death: only lower respiratory tract infections (LRTI), bloodstream infections, intra-abdominal infections, urinary tract infections and tuberculosis have higher AMR-attributable mortality. In Africa the burden is particularly high, reflected by SSTI leading to an estimated 16.2% of all adult inpatient antibiotic prescriptions for systemic use; the highest of proportion of any global region [7]. Versporten and colleagues found Africa to be the only regional setting where SSTI inpatient antibiotic prescriptions estimates exceed those for inpatient pneumonia/LRTI (10.3%), doing so by over 50%.

To date, the vast majority of microbiological data for infected wounds, SSTI and SSI in Africa are from small, single-centre cross-sectional studies. This systematic review and meta-analysis of proportions synthesises the data available from Central, Eastern, Southern and Western Africa over a ten-year period. It aims to report the extent of AMR in *S*. *aureus*, *E*. *coli*, *K*. *pneumoniae*, *P*. *aeruginosa* and *A*. *baumannii* to the antibiotic classes commonly used in clinical practice for empiric and targeted treatment.

## Materials and methods

### Literature search

The literature search was designed to understand: “what proportion of *S*. *aureus*, *E*. *coli*, *K*. *pneumoniae*, *P*. *aeruginosa* and *A*. *baumannii* isolates from infected wounds (including infected chronic wounds and ulcers), SSI and SSTI have been reported to be antimicrobial resistant in Central, Eastern, Southern and Western Africa in the last ten years?”. This question was summarised into three key concepts, restricted to publication from 1^st^ January 2012: Concept 1) Wounds, surgical sites, skin infections, burns and trauma; Concept 2) Antimicrobial resistance; and Concept 3) Central, Eastern, Southern and Western Africa.

The search was conducted in PubMed, MEDLINE and Embase to capture data from general patient populations (S1 Text). Duplicate articles were removed prior to further screening and assessment. The WHO’s African Index Medicus (AIM) was searched separately, using the search terms for the antimicrobial resistance concept alone, for additional eligible articles.

### Inclusion and exclusion criteria for studies

#### Study

Data from cross-sectional, cohort or case-control study designs were eligible for inclusion. Results from randomised controlled trials (RCT) were also included if they reported data from a control group or an intervention that did not impact upon bacterial isolate prevalence or resistance reporting. Case reports and case series were excluded to avoid the introduction of reporting bias. Studies had to be published between 1^st^ January 2012 and 15th August 2022, conducted in a healthcare facility within Central, Eastern, Southern or Western Africa, and available in English.

#### Population

There were no individual-level patient inclusion or exclusion criteria outside of the requirement for there to be a recorded clinical suspicion of wound infection, SSI or SSTI. Patients could be of any age and from any aspect of the healthcare system (primary, secondary or tertiary care as inpatients or outpatients). Data from cases of osteomyelitis were excluded, as this clinically distinct group of patients was not the focus of this review.

#### Outcomes

Studies had to report on 1) the prevalence of *S*. *aureus*, *E*. *coli*, *K*. *pneumoniae*, *P*. *aeruginosa* or *A*. *baumannii* in samples taken from infected wounds (including chronic wounds and ulcers), SSI and SSTI, and/or 2) the proportion of these isolates showing AMR to a prespecified list of agents within antibiotic classes (S2 Text) [8]. Species identification methods had to be described for all included studies. To be included for resistance data, phenotypic antibiotic sensitivity testing (manual or automated) or sensitivity testing methods using mass spectrometry had to be reported. Resistance mechanism data from genetic testing methods were not included.

#### Screening assessment

All identified articles were initially screened by title and abstract. Full-texts of successfully screened articles were then formally assessed to confirm suitability for inclusion. For both stages of screening and assessment, two authors considered articles independently, and a third author was consulted if consensus could not be reached.

#### Data extraction and handling

Study, patient and microbiological characteristics were extracted from all eligible studies. For each included organism of interest, the prevalence of isolation (stratified by type of infection) and the proportion of reported AMR was recorded. AMR definitions were species-specific, with antibiotic agents and classes chosen to reflect those used by the European Centre for Disease Control (ECDC) during the observation period of this review to determine multidrug resistance (MDR) [8].

#### Risk of bias assessment

All included studies were assessed for risk of bias according to the National Institutes of Health (NIH) Study Quality Assessment Tool for observational cohort and cross-sectional studies [9]. The five categories of potential bias were 1) population definition, 2) inclusion/exclusion clarity, 3) patient selection methods, 4) case establishment and 5) outcome establishment. Every study was assessed on each of these categories as low, medium or high risk of bias [10].

#### Determination of resistance within antibiotic classes

Resistance within an antibiotic class was defined as the proportion of non-susceptibility (isolates reported within studies as resistant, intermediate or non-susceptible) to at least one agent within the class [8]. Where non-susceptibility was reported within a single study to more than one agent within an antibiotic class, the agent with the higher proportion was used for further analysis. The proportions reported by studies were cross-referenced against the presented numerator and denominators where available: discrepant data were excluded from analysis.

#### Meta-analysis

Meta-analyses for proportions were performed using Stata version 15.1, to estimate the prevalence of each of the bacterial species of interest from infection samples. Analyses were stratified by infection type (infected wound, infected chronic wound/ulcer, SSI or SSTI). Analyses for the proportion of resistance observed within each antibiotic class were stratified by country. A sensitivity analysis was performed between prospective cohort studies and cross-sectional studies reporting data from SSI.

As studies reporting no resistance (0%) and complete resistance (100%) to an antibiotic class were included, a Freeman-Tukey double arcsine transformation was applied to each analysis in order to stabilise variances [11]. The 95% score confidence interval (CI) was used to calculate the CI of individual studies. A random-effects model with random-effects applied at the study-level was used to produce pooled estimates, with inverse-variance weighting used to account for differences in precision of individual study estimates. Pooled 95% CIs were calculated using the Wald method. A random-effects model was chosen over a fixed-effects model, given the expected variability of proportions between studies due to microbiological, population, and facility-level influences that could not be adjusted for by sub-group stratification.

## Results

### Results of the search

**Fig 1:**
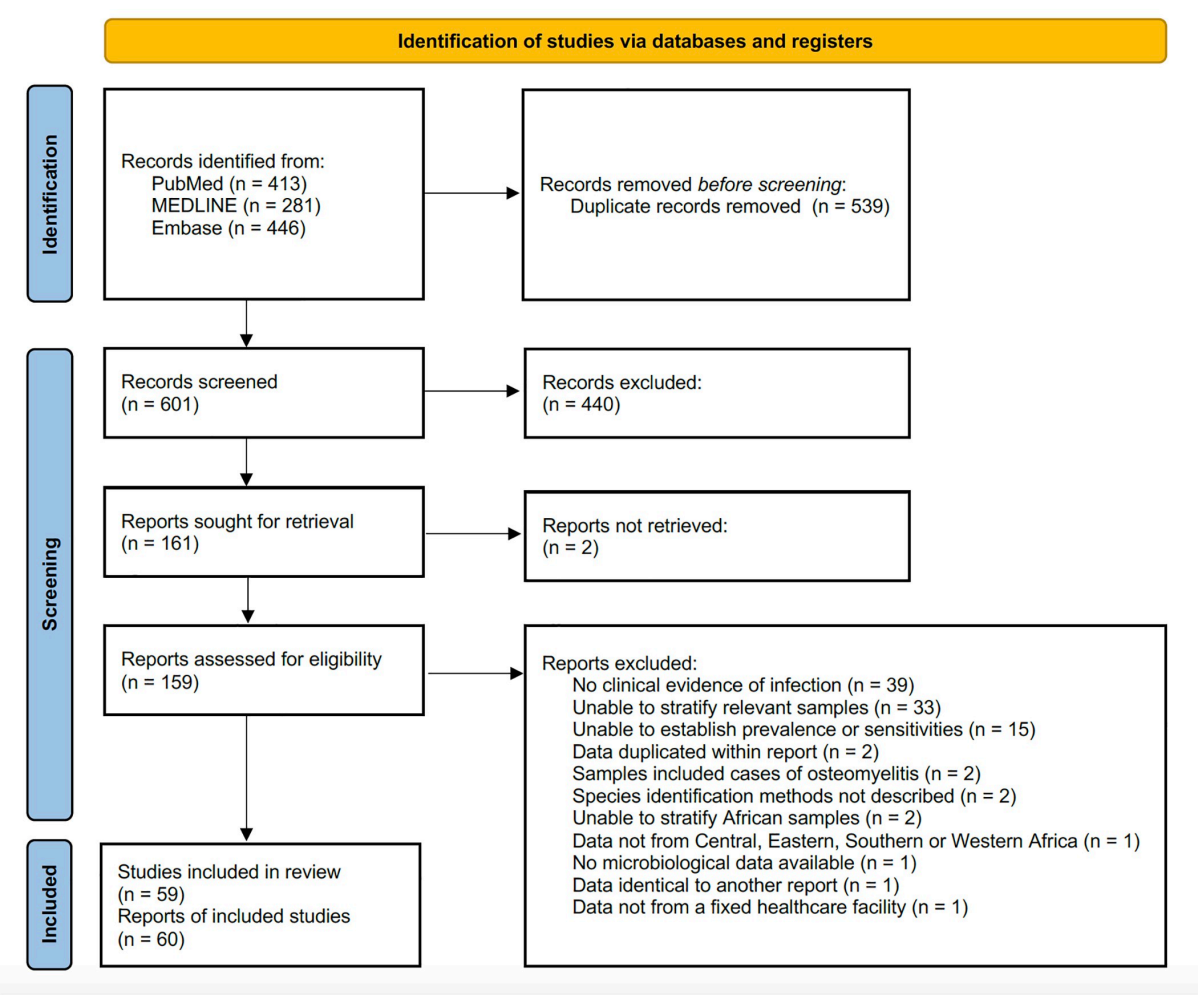
Literature search PRISMA flowchart.

The database search, conducted on 15^th^ August 2022, identified 601 unique articles (PubMed: 413, MEDLINE: 281, Embase: 446) that met the search criteria. No additional articles were found from the AIM search. After initial screening on title and abstract, the full-text of 161 articles were assessed for eligibility: 101 of these were subsequently excluded (Fig 1). The most common reasons for exclusion include: i) data were from patients without a clinical suspicion of infection (39/101: 39%), ii) data were from patients with infected wounds, SSI or SSTI not separated from other sample types (33/101: 33%), and iii) data were not presented to allow calculation of prevalence or antibiotic non-susceptibility (15/101: 15%). The remaining 60 articles reported data from 59 studies and are included in this review. Two articles reported data from the same study, these were extracted separately where the articles reported different aspects of the study, but data that was repeated was only recorded once [12,13].

#### Included studies

The study design, geographical, population and outcome reporting characteristics of the 59 studies included in this systematic review are summarised in Table 1 [12–71].

**Table 1:** Study, patient and outcome reporting characteristics of the studies included in this systematic review.

Study characteristics				Patient characteristics				Outcome reporting				
Study	Country	Study Design	First data collection (total months)	Age	Rural/ urban residence	Type of infection	Specific characteristics	SA	EC	KP	PA	AB
Abayneh 2022 [14]	Ethiopia	Prospective cohort	2021 (4)	Not reported	Mixed (52%/48%)	SSI		Prev Sens	Prev Sens	- -	Prev Sens	- -
Abosse 2020 [15]	Ethiopia	Cross-sectional	2019 (5)	Adults and children	Mixed (58%/42%)	SSI		Prev Sens	Prev Sens	- -	Prev Sens	Prev Sens
Adeyemo 2021 [16]	Nigeria	Cross-sectional	2016 (10)	Adults	Not reported	Chronic wounds/ulcers	Diabetic foot ulcers	Prev Sens	Prev -	- -	Prev -	- -
Akinloye 2021 [17]	Nigeria	Cross-sectional	2017 (13)	Adults	Not reported	Wounds		Prev -	Prev -	- -	Prev Sens	- -
Alebel 2021 [18]	Ethiopia	Cross-sectional	2020 (5)	Adults and children	Mixed (51%/49%)	Wounds	Patients in ITU	- -	Prev -	Prev -	Prev -	- -
Alelign 2022 [19]	Ethiopia	Cross-sectional	2021 (11)	Not reported	Mixed (57%/43%)	SSI	Orthopaedic surgery	Prev Sens	Prev Sens	Prev Sens	Prev Sens	Prev Sens
Bediako-Bowan 2020 [20]	Ghana	Prospective cohort	2017 (22)	Not reported	Not reported	SSI		Prev Sens	Prev Sens	Prev Sens	Prev Sens	Prev Sens
Bitew Kifilie 2018 [21]	Ethiopia	Cross-sectional	2016 (5)	Adults^†^	Mixed (22%/78%)	SSI	Caesarean section and episiotomy	Prev Sens	Prev Sens	Prev Sens	Prev Sens	- -
De Nardo 2016 [22]	Tanzania	Prospective cohort	2013 (3)	Adults^†^	Not reported	SSI	Caesarean section	Prev Sens	Prev -	- -	Prev -	- -
Desalegn 2020 [23]	Ethiopia	Cross-sectional	2019 (-)	Adults and children	Mixed (30%/70%)	SSTI	Dermatology outpatients	Prev Sens	- -	- -	- -	- -
Dessie 2016 [24]	Ethiopia	Prospective cohort	2013 (6)	Adults and children	Mixed (54%/46%)	SSI		Prev Sens	Prev Sens	Prev Sens	Prev Sens	- -
Egyir 2021 [25]	Ghana	Cross-sectional	2018 (6)	Adults and children	Not reported	SSI		Prev Sens	- -	- -	- -	- -
Garoy 2019 [26]	Eritrea	Cross-sectional	2017 (4)	Adults and children	Not reported	SSTI and wounds		Prev Sens	- -	- -	- -	- -
Garoy 2021 [27]	Eritrea	Cross-sectional	2016 (4)	Adults and children	Not reported	SSI		Prev Sens	Prev Sens	- -	- -	- -
Gemechu 2021 [28]	Ethiopia	Cross-sectional	2015 (9)	Adults	Not reported	SSI		Prev Sens	Prev Sens	Prev Sens	Prev -	- -
George 2018[29]	Uganda	Cross-sectional	2015 (5)	Adults and children	Rural	SSI		Prev Sens	Prev Sens	- -	Prev Sens	- -
Hope 2019[30]	Uganda	Cross-sectional	2015 (3)	Adults and children	Not reported	SSI		Prev Sens	Prev Sens	- -	- -	- -
Janssen 2018[31]	Ghana	Cross-sectional	2014 (5)	Adults and children	Rural	Wounds		Prev Sens	Prev Sens	Prev Sens	Prev Sens	Prev Sens
Kabanangi 2021[32]	Tanzania	Cross-sectional	2017 (10)	Children	Not reported	Wounds	Patients with burns	- -	Prev Sens	- -	Prev Sens	- -
Kahsay 2014[33]	Ethiopia	Cross-sectional	2011 (4)	Adults	Mixed (65%/35%)	SSI		Prev Sens	- -	- -	- -	- -
Kalayu 2019[34]	Ethiopia	Prospective cohort	2016 (13)	Adults and children	Not reported	SSI		Prev Sens	Prev Sens	- -	- -	- -
Kassam 2017[35]	Tanzania	Cross-sectional	2013 (12)	Adults and children	Not reported	Wounds		Prev Sens	Prev Sens	Prev Sens	Prev Sens	- -
Kazimoto 2018[36]	Tanzania	Cross-sectional	2012 (9)	Adults and children	Not reported	SSTI		Prev Sens	Prev -	Prev Sens	Prev Sens	Prev -
Khalim 2021[37]	Uganda	Cross-sectional	2020 (3)	Adults and children	Not reported	Chronic wounds/ulcers		Prev -	- -	- -	Prev -	- -
Krumkamp 2020[38]	Ghana	Cross-sectional	2016 (11)	Adults	Rural	Chronic wounds/ulcers		Prev Sens	Prev -	Prev Sens	Prev -	- -
Lakoh 2022[39]	Sierra Leone	Prospective cohort	2021 (6)	Adults	Urban	SSI		- -	Prev -	Prev -	- -	Prev -
Mama 2014[40]	Ethiopia	Cross-sectional	2013 (5)	Adults and children	Not reported	Wounds		Prev Sens	Prev Sens	Prev Sens	Prev Sens	- -
Mama 2019[41]	Ethiopia	Cross-sectional	2017 (3)	Adults and children	Mixed (40%/60%)	SSI and wounds		Prev Sens	- -	- -	- -	- -
Manyahi 2014[42]	Tanzania	Cross-sectional	2011 (5)	Adults	Not reported	SSI		Prev Sens	Prev Sens	Prev Sens	Prev Sens	Prev Sens
Mekonnen 2021[43]	Ethiopia	Cross-sectional	2020 (3)	Adults and children	Mixed (62%/38%)	SSI		- -	- -	- -	Prev -	- -
Mengesha 2014 [44]	Ethiopia	Cross-sectional	2012 (6)	Adults	Not reported	SSI		Prev Sens	Prev Sens	- -	Prev Sens	- -
Misha 2021 [45]	Ethiopia	Prospective cohort	2019 (5)	Adults	Mixed (73%/27%)	SSI		Prev Sens	Prev Sens	- -	Prev Sens	- -
Moges 2019 [46]	Ethiopia	Cross-sectional	2017 (5)	Adults and children	Mixed (59%/41%)	Wounds		- -	- -	Prev Sens	- -	Prev Sens
Mohammed 2013 [47]	Nigeria	Cross-sectional*	2010 (3)	Not reported	Mixed (63%/37%)	Wounds		Prev Sens	Prev Sens	- -	Prev Sens	- -
Mohammed 2017 [48]	Ethiopia	Cross-sectional	2014 (3)	Adults and children	Not reported	Wounds		Prev Sens	Prev Sens	- -	Prev Sens	- -
Monnheimer 2021 [49]	Ghana	Cross-sectional	2017 (8)	Not reported	Rural	SSI and acute/ chronic wounds		- -	- -	- -	- -	Prev -
Moremi 2019 [50]	Tanzania	Prospective cohort	2014 (10)	Not reported	Not reported	SSI		Prev Sens	- -	- -	- -	- -
Motbainor 2020 [51]	Ethiopia	Cross-sectional	2018 (4)	Adults and children	Mixed (47%/53%)	SSI		- -	- -	- -	Prev -	Prev -
Muhindo 2021 [52]	Uganda	Cross-sectional	2016 (9)	Not reported	Not reported	SSI		- Sens	- Sens	- Sens	- Sens	- -
Mukagendaneza 2019 [53]	Rwanda	Prospective cohort	2017 (4)	Adults	Not reported	SSI		Prev -	Prev -	- -	- -	- -
Nwankwo 2014 [54]	Nigeria	Cross-sectional	2009 (24)	Adults and children	Not reported	SSI		Prev Sens	Prev Sens	Prev Sens	Prev Sens	- -
Oladeinde 2013 [55]	Nigeria	Cross-sectional	2006 (48)	Adults and children	Rural	Wounds		Prev Sens	Prev Sens	- -	Prev Sens	- -
Omer 2020 [56]	Sudan	Cross-sectional	2016 (1)	Not reported	Not reported	Chronic wounds/ulcers	Patients with diabetes	- -	- -	- -	Prev Sens	- -
Pondei 2013 [57]	Nigeria	Cross-sectional*	2020 (4)	Adults and children	Not reported	Wounds		Prev Sens	Prev Sens	- -	Prev Sens	- -
Rafai 2015 [58]	Central African Republic	Cross-sectional	2011 (13)	Adults and children	Not reported	SSI		Prev Sens	Prev -	Prev -	Prev Sens	Prev Sens
Seni 2013 [12,13]	Uganda	Cross-sectional	2011 (8)	Adults and children	Not reported	SSI		Prev Sens	Prev Sens	- -	Prev Sens	- -
Shakir 2021 [59]	Ethiopia	Prospective cohort	2020 (1)	Adults and children	Mixed (48%/52%)	SSI		Prev -	Prev -	- -	- -	- -
Shimekaw 2020 [60]	Ethiopia	Cross-sectional	2019 (5)	Adults and children	Mixed (55%/45%)	Wounds		Prev Sens	Prev Sens	Prev Sens	Prev Sens	- -
Tadesse 2018 [61]	Ethiopia	Cross-sectional	2013 (7)	Adults and children	Not reported	SSI		Prev Sens	- -	- -	- -	- -
Tambuwal 2020 [62]	Nigeria	Cross-sectional	2014 (6)	Adults and children	Not reported	SSTI		- Sens	- -	- -	- -	- -
Tefera 2021 [63]	Ethiopia	Cross-sectional	2020 (3)	Adults and children	Mixed (71%/29%)	SSI		Prev Sens	- -	- -	- -	- -
Tilahun 2022 (1) [64]	Ethiopia	Cross-sectional	2021 (7)	Adults and children	Mixed (61%/39%)	Wounds		- -	- -	- -	Prev -	- -
Tilahun 2022 (2) [65]	Ethiopia	Cross-sectional	2021 (11)	Adults and children	Mixed (24%/76%)	SSI		Prev Sens	Prev Sens	Prev Sens	- -	- -
Tsige 2020 [66]	Ethiopia	Cross-sectional	2016 (3)	Adults and children	Mixed (23%/77%)	Wounds		Prev Sens	- -	- -	- -	- -
Van der Meeren 2013 [67]	Mozambique	Cross-sectional	2010 (10)	Adults and children	Not reported	SSTI and wounds		Prev Sens	- -	- -	- -	- -
Velin 2021 [68]	Rwanda	Prospective cohort	2019 (6)	Adults^†^	Rural	SSI	Caesarean section	Prev Sens	Prev Sens	Prev Sens	Prev Sens	Prev Sens
Wangoye 2022 [69]	Uganda	Cross-sectional	2020 (3)	Not reported	Not reported	Chronic wounds/ulcers		Prev -	- -	- -	Prev -	- -
Wekesa 2020 [70]	Uganda	Cross-sectional	2017 (6)	Adults^†^	Not reported	SSI	Caesarean section	Prev Sens	Prev Sens	- -	Prev Sens	- -
Yagoup 2019 [71]	Sudan	Cross-sectional	2016 (19)	Adults and children	Not reported	Wounds		- -	- -	- -	Prev	- -

*Cases selected from laboratory samples, rather than from patient cohort.

^†^Women of childbearing age. ITU = intensive care unit, SSI = surgical site infections, SSTI = skin and soft tissue infections, SA = *Staphylococcus aureus*, EC = *Eschericheria coli*, KP = *Klebsiella pneumoniae*, PA = *Pseudomonas aeruginosa*, AB = *Acinetobacter baumannii*, Prev = prevalence, Sens = sensitivity.

### Types of study

The most common study design was cross-sectional (48/59: 81%), whilst the remaining 11 studies were prospective cohort studies. Of the cross-sectional studies, 46/48 (96%) recruited patients directly from clinical settings, whereas two studies utilised samples from laboratory storage which were specifically reported to have come from patients with clinical signs of an infected wound, SSI or SSTI.

### Time period and geographical location

Of the 58 studies that reported their dates and duration of observation, the median start date of observation was 2017 (interquartile range [IQR] 2013–2019), with a median observation duration of six months (IQR 4–9.5).

Studies included data from 11 countries, including countries from Central Africa (Central African Republic [n = 1] and Rwanda [n = 2]), Eastern Africa (Eritrea [n = 2], Ethiopia [n = 25], Sudan [n = 2], Tanzania [n = 6] and Uganda [n = 7]), Southern Africa (Mozambique [n = 1]) and Western Africa (Ghana [n = 5], Nigeria [n = 7] and Sierra Leone [n = 1]).

### Population and sampling site

Of the studies included, 35/59 (59%) reported on data from both children and adults. Fourteen studies (24%) only included adults and one study (2%) only children. The remaining studies (9/59: 15%) did not report age. Of the 27 studies declaring their population’s rural/urban distribution, 6/27 (30%) had an entirely rural population and 1/27 (5%) entirely urban: 13/20 (65%) of studies with a mixed population were predominantly rural. Study-specific inclusion criteria or wound characteristics are shown in Table 1.

### Outcome measures reported

Universally, samples were processed in the same laboratories which provided the routine microbiology diagnostics for the recruiting healthcare facility, using local culture procedures and methods for direct sensitivity testing (S1 Table). Techniques for species identification included a combination of colony morphological interpretation, Gram stain and various biochemical tests according to local practices in the majority (56/59: 95%) of studies. Of these, eight (14%) used an API system, MALDI biotyper (Bruker Daltronics, Bremen, Germany) or VITEK 2/VITEK 2 Compact (*bioMerieux*, Marcy l’Etoile, France) to confirm indeterminate/all isolates and five (9%) included them in their methods but not systematically. In three studies (5%), morphological interpretation, Gram stain and biochemical tests were not performed and identification was established with a MALDI biotyper or VITEK 2/VITEK 2 Compact system only.

The prevalence of *S*. *Aureus* was reported in 47/59 (80%) studies and resistance within an antibiotic class of importance was reported in 44/59 (75%): for *E*. *coli*, 38/59 (64%) studies reported prevalence and 29/59 (49%) reported resistance; for *K*. *pneumoniae*, 19/59 (32%) studies reported prevalence and 17/59 (29%) reported resistance; for *P*. *aeruginosa*, 38/59 (64%) studies reported prevalence and 28/59 (47%) reported resistance; and for *A*. *baumannii*, 12/59 (20%) studies reported prevalence and 8/59 (14%) reported resistance.

### Funding

Local funding was reported for a significant proportion of studies: 17/59 (29%) of studies were funded by affiliated universities and 3/59 (5%) were funded by the local Ministry of Health. Bilateral donors funded 8/59 (14%) of studies and 2/59 (3%) of the studies were funded by multilateral donors (NGOs). 13/59 (22%) studies received no funding, whilst 16/59 (27%) did not state

### Risk of bias assessment

**Fig 2:**
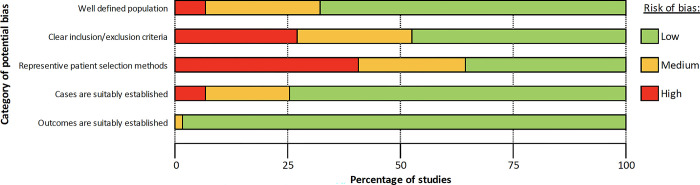
Risk of bias assessment summary.

We classed most studies as having low or moderate overall risk of bias (Fig 2). The most substantial risk of bias identified across all studies was from patient selection methods, with 24/59 (41%) determined as having a high risk, followed by the inclusion and exclusion criteria reported in the studies (16/59 [27%]). The category of potential bias in which the studies performed the best was how outcomes were established, with 58/59 (98%) studies having a low risk (S3 Text).

### Prevalence of organisms isolated from samples

**Fig 3:**
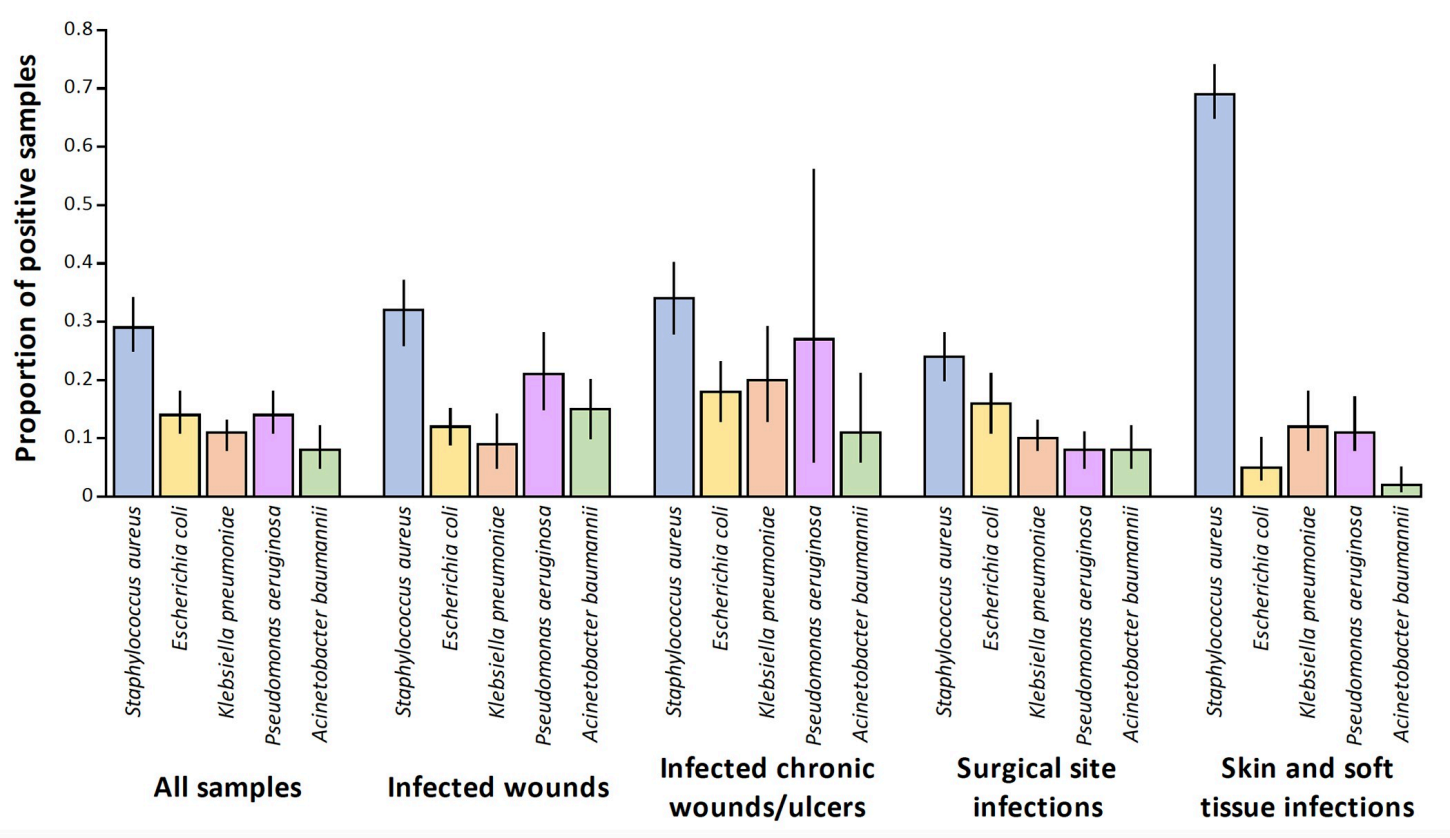
Proportion of samples positive for *Staphylococcus aureus*, *Escherichia coli*, *Klebsiella pneumoniae*, *Pseudomonas aeruginosa* and *Acinetobacter baumannii*, according to infection type (with 95% confidence intervals) in Central, Eastern, Southern and Western Africa. Bars represent pooled proportions and error-bars the 95% confidence intervals.

When considering all relevant samples from contributing studies through pooled meta-analysis of proportions, *S*. *aureus* was isolated in 29% (95% confidence interval [CI] 25% to 34%: 47 studies) of samples, *E*. *coli* in 14% (CI 11% to 18%: 38 studies), *K*. *pneumoniae* in 11% (CI 8% to 13%, 19 studies), *P*. *aeruginosa* in 14% (CI 11% to 18%: 38 studies) and *A*. *baumannii* in 8% (CI 5% to 12%: 12 studies) (Fig 3).

*S*. *aureus* was the most commonly isolated organism across all types of infection: prevalence was highest in SSTI (69%, CI 65% to 74%: four studies) and similar in all remaining types of infection: 32% in infected wounds (CI 26% to 37%: 12 studies), 34% in infected chronic wounds and ulcers (CI 28% to 40%, four studies) and 24% in SSI (CI 20% to 28%: 30 studies). Of the Gram negative bacilli, *P*. *aeruginosa* was more commonly isolated from infected wounds (21%, CI 15% to 28%: 13 studies) than *E*. *coli* (12%, CI 9% to 15%: 11 studies) or *K*. *pneumoniae* (9%, CI 5% to 14%: six studies).

### Antimicrobial resistance estimates

The rates of resistance observed to agents within the antibiotic classes of interest in isolates of *S*. *aureus*, *E*. *coli*, *K*. *pneumoniae*, *P*. *aeruginosa* and *A*. *baumannii* are presented in Table 2. Regional AMR estimates are included in the Supporting Information (S2 Table).

**Table 2:** Pooled estimates of antimicrobial resistance in wound, skin, soft tissue and surgical site infections in Central, Eastern, Southern and Western Africa.

Antibiotic class (agents)	Resistance	95% Confidence Interval	Data source	I^2^
***Staphylococcus aureus***				
Aminoglycosides	0.24	0.15 to 0.34	1422 samples	94%
(Gentamicin)			(29 studies)	
Ansamycins	0.08	0.00 to 0.28	183 samples	87%
(Rifampin)			(3 studies)	
Anti-staphylococcal beta-lactams/cephamycins	0.48	0.35 to 0.61	1611 samples	96%
(Cefoxitin, methicillin or oxacillin)			(32 studies)	
Fluoroquinolones	0.19	0.13 to 0.26	1485 samples	89%
(Ciprofloxacin)			(23 studies)	
Folate synthesis inhibitors	0.53	0.37 to 0.70	1432 samples	97%
(Trimethoprim-sulphamethoxazole)			(27 studies)	
Glycopeptide	0.03	0.00 to 0.09	750 samples	89%
(Vancomycin)			(16 studies)	
Lincosamides	0.24	0.12 to 0.37	1159 samples	95%
(Clindamycin)			(22 studies)	
Macrolides	0.44	0.33 to 0.56	1353 samples	94%
(Erythromycin)			(27 studies)	
Oxazolidinones	0	N/A	33 samples	N/A
(Linezolid)			(1 study)	
Phenicols	0.36	0.16 to 0.60	1023 samples	98%
(Chloramphenicol)			(17 studies)	
Phosphoric acids	0	NA	31 samples	N/A
(Fosfomycin)			(1 study)	
Tetracyclines	0.54	0.40 to 0.67	1392 samples	96%
(Doxycycline or tetracycline)			(26 studies)	
***Escherichia coli***				
Aminoglycosides	0.51	0.42 to 0.60	609 samples	73%
(Amikacin or gentamicin)			(23 studies)	
Anti-pseudomonal penicillins with beta-lactamase inhibitors	0.48	0.27 to 0.70	249 samples	89%
(Piperacillin-tazobactam)			(4 studies)	
Carbapenems	0.06	0.00 to 0.19	418 samples	90%
(Imipenem or meropenem)			(13 studies)	
First/second generation cephalosporins	0.72	0.58 to 0.85	213 samples	62%
(Cefazolin or cefuroxime)			(6 studies)	
Third/fourth generation cephalosporins	0.74	0.60 to 0.86	727 samples	91%
(Cefepime, cefotaxime, ceftazidime or ceftriaxone)			(25 studies)	
Cephamycins	0.52	0.32 to 0.71	131 samples	71%
(Cefoxitin)			(5 studies)	
Fluoroquinolones	0.54	0.40 to 0.66	831 samples	91%
(Ciprofloxacin)			(24 studies)	
Folate pathway inhibitors	0.83	0.73 to 0.92	732 samples	86%
(Trimethoprim-sulphamethoxazole)			(20 studies)	
Penicillins	0.93	0.88 to 0.98	764 samples	75%
(Ampicillin)			(22 studies)	
Penicillins with beta-lactamase inhibitors	0.8	0.66 to 0.92	729 samples	92%
(Amoxicillin-clavulanic acid)			(18 studies)	
Phenicols	0.51	0.33 to 0.68	387 samples	91%
(Chloramphenicol)			(15 studies)	
Tetracyclines	0.81	0.71 to 0.91	403 samples	79%
(Doxycycline or tetracycline)			(17 studies)	
***Klebsiella pneumoniae***				
Aminoglycosides	0.38	0.20 to 0.57	201 samples	85%
(Amikacin or gentamicin)			(12 studies)	
Anti-pseudomonal penicillins with beta-lactamase inhibitors	0.58	0.44 to 0.71	57 samples	1%
(Piperacillin-tazobactam)			(3 studies)	
Carbapenems	0.08	0.00 to 0.24	262 samples	90%
(Imipenem or meropenem)			(9 studies)	
First/second generation cephalosporins	0.66	0.23 to 0.98	102 samples	95%
(Cefazolin or cefuroxime)			(5 studies)	
Third/fourth generation cephalosporins	0.61	0.41 to 0.80	256 samples	88%
(Cefepime, cefotaxime, ceftazidime or ceftriaxone)			(14 studies)	
Cephamycins	0.43	0.10 to 0.79	57 samples	86%
(Cefoxitin)			(3 studies)	
Fluoroquinolones	0.32	0.15 to 0.52	254 samples	89%
(Ciprofloxacin)			(13 studies)	
Folate pathway inhibitors	0.78	0.62 to 0.90	261 samples	84%
(Trimethoprim-sulphamethoxazole)			(14 studies)	
Penicillins with beta-lactamase inhibitors	0.85	0.69 to 0.96	180 samples	81%
(Amoxicillin-clavulanic acid)			(9 studies)	
Phenicols	0.51	0.28 to 0.74	99 samples	80%
(Chloramphenicol)			(6 studies)	
Tetracyclines	0.69	0.51 to 0.85	143 samples	78%
(Doxycycline or tetracycline)			(10 studies)	
***Pseudomonas aeruginosa***				
Aminoglycosides	0.19	0.09 to 0.32	590 samples	88%
(Amikacin or gentamicin)			(25 studies)	
Anti-pseudomonal carbapenems	0.21	0.02 to 0.49	404 samples	97%
(Imipenem or meropenem)			(15 studies)	
Anti-pseudomonal cephalosporins	0.41	0.22 to 0.61	600 samples	94%
(Cefepime or ceftazidime)			(20 studies)	
Antipseudomonal fluoroquinolones	0.26	0.14 to 0.42	670 samples	92%
(Ciprofloxacin)			(25 studies)	
Anti-pseudomonal penicillins with beta-lactamase inhibitors	0.28	0.02 to 0.64	57 samples	86%
(Piperacillin-tazobactam)			(3 studies)	
Monobactams	0.88	0.81 to 0.93	122 samples	0%
(Aztreonam)			(2 studies)	
Polymyxins	0.25	0.16 to 0.36	69 samples	NA
(Polymyxin B)			(1 study)	
***Acinetobacter baumannii***				
Aminoglycosides	0.57	0.37 to 0.76	70 samples	57%
(Amikacin or gentamicin)			(6 studies)	
Anti-pseudomonal carbapenems	0.2	0.09 to 0.34	81 samples	40%
(Imipenem or meropenem)			(7 studies)	
Anti-pseudomonal fluoroquinolones	0.45	0.30 to 0.60	47 samples	0%
(Ciprofloxacin)			(5 studies)	
Anti-pseudomonal penicillins with beta-lactamase inhibitors	0.03	0.00 to 0.17	29 samples	0%
(Piperacillin-tazobactam)			(2 studies)	
Extended-spectrum cephalosporins	0.7	0.17 to 1.00	71 samples	93%
(Cefepime, cefotaxime, ceftazidime or ceftriaxone)			(7 studies)	
Folate pathway inhibitors	0.67	0.39 to 0.90	15 samples	0%
(Trimethoprim-sulphamethoxazole)			(2 studies)	
Tetracyclines	0.49	0.08 to 0.90	62 samples	46%
(Doxycycline or tetracycline)			(5 studies)	

### Staphylococcus aureus

Average pooled anti-staphylococcal beta-lactam/cephamycin resistance (cefoxitin, methicillin or oxacillin), suggesting methicillin-resistant *S*. *aureus* (MRSA), was 48% (CI 35% to 61%: 32 studies). Regional differences in resistance were observed across a number of antibiotic classes for *S*. *aureus*. Anti-staphylococcal beta-lactam/cephamycin resistance, indicating MRSA, was lower in studies conducted in Western Africa (23%, CI 9% to 41%: six studies) compared to Eastern Africa (58%, CI 41% to 73%: 23 studies). A higher resistance to lincosamides was also observed in Eastern Africa (32%, CI 17% to 49%: 16 studies) compared to other regions (9%, CI 5% to 16% in Central Africa: one study; 2%, CI 1% to 7% in Southern Africa: one study; and 6%, CI 1% to 13% in Western Africa: four studies).

### Enterobacteriaceae

In *E*. *coli*, AMR was estimated to be higher than 40% for all antibiotic classes apart from carbapenems (6%, CI 0% to 19%: 13 studies). A similar pattern of resistance was seen in *K*. *pneumoniae*, which had a pooled estimate of over 30% resistance to all antibiotic classes apart from carbapenems (8%, CI 0% to 24%: nine studies). Significant variation was seen between regions for carbapenem resistance, which was high in Eastern Africa (10%, CI 0% to 28% for *E*. *coli*: 10 studies, and 31%, CI 5% to 64% for *K*. *pneumoniae*: four studies) and low in all other regions.

### Pseudomonads

The pooled rates of resistance observed to agents within the antibiotic classes of interest in isolates of *P*. *aeruginosa* and *A*. *baumannii* were all 20% or greater, apart from aminoglycosides for *P*. *aeruginosa* (19%, CI 9% to 32%: 25 studies) and anti-pseudomonal penicillins with beta-lactamase inhibitors for *A*. *baumannii* (3%, CI 0% to 17%: two studies). Significant variation was seen between regions for resistance to anti-pseudomonal penicillins with beta-lactamase inhibitors, which was high in Eastern Africa (45%, CI 29% to 60% for *P*. *aeruginosa*: two studies, and 50%, CI 19% to 81% for *A*. *baumannii*: one study) and low in Western Africa (10%, CI 3% to 30% for *P*. *aeruginosa*: one study, and 0%, CI 0% to 14% for *A*. *baumannii*: one study).

### Sensitivity analysis

The impact of the potential bias introduced by selective sampling of more critically ill patients was explored in a sensitivity analysis, comparing the AMR reported in SSI prospective cohort studies, which assessed for the development of SSI in well patients prior to starting antibiotics, and cross-sectional SSI studies, which were more likely to include patients failing treatment. Similar patterns of AMR were estimated between these two types of study (S3 Table).

## Discussion

This review reports the isolation of *S*. *aureus*, *E*. *coli*, *K*. *pneumoniae*, *P*. *aeruginosa* and *A*. *baumannii* from the majority of swabs taken from clinically infected wounds, skin, soft tissue and surgical sites in Central, Eastern, Southern and Western Africa: *S*. *aureus* was the most commonly isolated organism, particularly in SSTI. AMR was high across all five species: of particular note, *S*. *aureus* was resistant to methicillin (MRSA) in >40% of isolates; *E*. *coli* and *K*. *pneumoniae* were both resistant to amoxicillin-clavulanic acid in ≥80% of isolates and resistant to aminoglycosides in 51% and 38% of isolates respectively; and *P*. *aeruginosa* and *A*. *baumannii* were both resistant to anti-pseudomonal carbapenems (imipenem or meropenem) in ≥20% of isolates. All of these resistant isolates have the potential to represent priority pathogens requiring critical (extended spectrum beta-lactamase [ESBL]-producing or carbapenem-resistant *Enterobacteriaceae*, carbapenem-resistant *P*. *aeruginosa* and carbapenem-resistant *A*. *baumannii*) or urgent (MRSA) development of new antibiotics according to the WHO [72]. It is essential however, that plans for antimicrobial development are accompanied by policies that ensure equitable access once available.

The majority of the current literature considering wound infections in Africa is summarised in two recent systematic reviews from Ethiopia by Chelkeba et al. [5,6]. They found similar results in wound infections to our systematic review, with *S*. *aureus* being the bacterial pathogen most commonly isolated (36%) and *E*. *coli*, *K*. *pneumoniae*, *P*. *aeruginosa* and *A*. *baumannii* all contributing to a smaller proportion of infections (8–17%). Our systematic review covers a larger geographical area, stratifies by types of infection (clinically infected wounds, SSTI, and SSI), and focusses on clinically relevant isolates only. Other systematic reviews of AMR in Africa, not restricted to wound infection, SSI and SSTI, reported similar levels of MRSA amongst isolates, but much higher levels of vancomycin resistance [73,74], whereas our systematic review suggested a pooled vancomycin resistance estimate of only 3% (CI 0% to 9%: 16 studies). This may be due to previous reviews including samples taken to screen for carriage of resistant organisms in healthy individuals.

Compared to Murray et al’s recent review on global AMR burden, we found higher third generation cephalosporin resistance in *E*. *coli*, similar to that reported in Southeast Asia where resistance is almost uniformly over 50% [4]. This might reflect the proportion of patients with surgical site infections in our review and the use of ceftriaxone in surgical prophylaxis, selecting out isolates resistant to third generation cephalosporins. Our results were consistent to those estimates by Murray and colleagues with respect to the proportion of MRSA amongst *S*. *aureus* isolates, levels of third generation cephalosporin resistance in *K*. *pneumoniae*, and carbapenem levels of resistance across the Gram negative organisms [4].

This systematic review also highlights the microbiological diagnostic gaps in many African clinical settings. A recent review found Africa to be the global region with the lowest proportion of available AMR national action plans (NAPs) [75]. This may explain why we only found data for 11 countries in Central, Eastern, Southern and Western Africa, albeit using English search terms: whilst very few studies where screened out due to language, our search may have missed articles published in other languages and therefore under-represent non-anglophone communities.

Sustainable, coordinated funding is an essential component of research and development, and it is notable that the funding for the studies included in this review were rarely awarded by international grants or large organisations: when obtained, the vast majority were supported by local universities attached to a tertiary healthcare facility, while over 20% of the included studies reported that they received no dedicated funding. AMR surveillance, diagnostic stewardship and targeted infection treatment would all benefit from strengthening local capacity with funding support, which would create an opportunity for local and regional standardisation, external quality assessment and reporting protocols, strengthening research and improving data quality [76]. In 2018, the WHO published it’s model list of essential in vitro diagnostics (EVD) that, much like its model list of essential medications, allows an objective analysis of knowledge gaps and provides a benchmark for laboratory practice [77,78]. These diagnostics are still lacking in many African healthcare settings [79].

Our review comprehensively collected clinical, microbiological and study design characteristics, allowing a detailed reporting of *S*. *aureus*, *E*. *coli*, *K*. *pneumoniae*, *P*. *aeruginosa* and A. *baumannii* AMR using data from almost 5,000 significant isolates from over 26,000 clinical samples. We also only included studies that presented data from patients with clinical signs of infection. Whilst contamination of samples from clinically infected wounds, skin, soft tissue or surgical sites is difficult to exclude, these methods will have increased the yield of clinically significant microbiological reporting.

One of the limitations of this review is that in settings with constrained microbiology resources, sampling might be limited to the most critically ill patients who are not responding to empirical treatment, applying selection pressure for resistant organisms that could lead to AMR over-reporting. However, our sensitivity analysis comparing prospective (following up all patients regardless of illness severity) and cross-sectional analyses did not find a difference. Another potential impact of limited resources is that access to reliable and timely microbiological reporting allows for improved antimicrobial stewardship, surveillance and infection prevention control. These might explain the disparities in MRSA prevalence between countries in this review.

The proportion of resistance reported to each antibiotic was not at isolate-level. Antibiograms, and thus resistance mechanisms and rates of multi-drug resistance (MDR), were not able to be established. Each study reported AMR to various antimicrobial agents/categories according to local protocols or the study design, rather than a standardised list. Therefore, the data presented cannot be used to establish optimal empirical therapies. They can, however, be used to estimate an approximate risk of failure of commonly used antimicrobial antibiotics.

Finally, the clinical settings included in this review varied by geography, time and patient characteristics, likely contributing to considerable heterogeneity. Whilst some of that heterogeneity could be explained by infection type and African sub-region, study-level population differences persisted in our sub-group analyses, indicated by high I^2^ statistics. It is also important to note that a large number of included studies were conducted in Ethiopia, and therefore potentially skewed by any prescribing practices, clinical guidelines or diagnostic access specific to the country.

## Conclusion

The prevalence of AMR in *S*. *aureus*, *E*. *coli*, *K*. *pneumoniae*, *P*. *aeruginosa* and *A*. *baumannii* isolates from clinically infected wounds, skin, soft tissue and surgical sites in Central, Eastern, Southern and Western African healthcare settings was high. In particular, a large proportion of these organisms display resistance patterns with critical or urgent WHO priority for antimicrobial development. We highlight significant gaps in microbiological testing capacity in Africa, with contributions from only 11 countries: a strong signal that we are far from diagnostic equity and the WHO’s EVD goals. Achieving reliable, timely testing and reporting of microbiological samples is essential to 1) benefit patient care through more informed empiric treatments regimens and more frequently targeted therapy, 2) improve surveillance, 3) support diagnostic stewardship practices and 4) allow standardisation for quality assurance and academic dissemination of higher quality, comparable data.

## Supporting information

S1 Checklist PRISMA checklist. (DOCX)

S1 Text Literature search terms. (DOCX)

S2 Text Antibiotic agents and classes of interest according to species. (DOCX)

S3 Text Study-level risk of bias assessment. (DOCX)

S1 Table Methods of bacterial species identification and susceptibility testing. (DOCX)

S2 Table Regional AMR estimates. (DOCX)

S3 Table SSI prospective cohort study versus cross-sectional study sensitivity analysis. (DOCX)
