# Correction: Antimicrobial resistance in bacterial wound, skin, soft tissue and surgical site infections in Central, Eastern, Southern and Western Africa: A systematic review and meta-analysis

**DOI:** 10.1371/journal.pgph.0004608

**Published:** 2025-05-09

**Authors:** Edward J. M. Monk, Timothy P. W. Jones, Felix Bongomin, Winnie Kibone, Yakobo Nsubuga, Nelson Ssewante, Innocent Muleya, Lauryn Nsenga, V. Bhargavi Rao, Kevin van Zandvoort

There is an error in affiliation 6 for authors Innocent Muleya and V. Bhargavi Rao. The correct affiliation 6 is: MSF UK, London, United Kingdom.

There is an error in the third subheading of Antimicrobial resistance estimates in the Results. The correct subheading is: *Pseudomonadales*.

There are multiple errors in the hierarchy of the sections and subsections introduced during copyediting.

In the Materials and methods, the Screening assessment, Data extraction and handling, Risk of bias assessment, Determination of resistance within antibiotic classes, and Meta-analysis subsections should be subsections of the Materials and methods.

In the Results, the Included studies subsection should be a subsection of the Results.

In the Results, the Types of study, Time period and geographical location, Population and sampling site, Outcome measures reported, and Funding subsections should be subsections of the Included Studies.

In the Results, the *Staphylococcus aureus*, *Enterobacteriaceae*, and *Pseudomonadales* subsections should be subsections of the Antimicrobial resistance estimates and their subheadings should be italicized.
