# Supplementary material for: Antimicrobial resistance in bacterial wound, skin, soft tissue and surgical site infections in Central, Eastern, Southern and Western Africa: A systematic review and meta-analysis
Source: PLOS Glob Public Health. 2024 Apr 16;4(4):e0003077. doi: 10.1371/journal.pgph.0003077 (PMC11020607; doi:10.1371/journal.pgph.0003077)
Supplement: S1 Text — (DOCX) [file pgph.0003077.s002.docx]

**S1 Text: Literature search terms**

| Concept | Search Terms |
| --- | --- |
| Wounds, surgical sites, skin infections, burns and trauma | (("skin"[Title/Abstract]) OR ("soft tissue*"[Title/Abstract]) OR ("wound*"[Title/Abstract]) OR ("burn*"[Title/Abstract]) OR ("trauma*"[Title/Abstract]) OR ("cellulitis"[Title/Abstract]) OR ("necrotising"[Title/Abstract]) OR ("necrotizing"[Title/Abstract])) OR ("surgical site*"[Title/Abstract])) |
|  | AND |
| Antibiotic resistance | (("microbial drug resistan*"[Title/Abstract]) OR ("bacterial resistan*"[Title/Abstract]) OR ("antibiotic resistan*"[Title/Abstract]) OR ("antimicrobial resistan*"[Title/Abstract]) OR ("XDR"[Title/Abstract]) OR ("MDR"[Title/Abstract]) OR ("multi-drug resistan*"[Title/Abstract]) OR ("drug resistan*"[Title/Abstract]) OR ("drug-resistan*"[Title/Abstract]) OR ("beta-lactamase*"[Title/Abstract]) OR ("carbapenamase*"[Title/Abstract]) OR ("ESBL"[Title/Abstract]) OR ("CTX-M"[Title/Abstract]) OR ("AmpC"[Title/Abstract]) OR ("metallo-beta-lactamase*"[Title/Abstract]) OR ("MBL"[Title/Abstract]) OR ("methicillin-resistant Staphylococcus aureus"[Title/Abstract]) OR ("MRSA"[Title/Abstract]) OR ("vancomycin-resistant Staphylococcus aureus"[Title/Abstract]) OR ("VRSA"[Title/Abstract])) |
|  | AND |
| Central, Eastern, Southern and Western Africa | ((“Africa*"[Title/Abstract]) OR (“sub-Sahara*"[Title/Abstract]) OR (“Angola"[Title/Abstract]) OR (“Benin"[Title/Abstract]) OR (“Botswana"[Title/Abstract]) OR (“Burkina Faso"[Title/Abstract]) OR (“Burundi"[Title/Abstract]) OR (“Cabo Verde"[Title/Abstract]) OR (“Cape Verde"[Title/Abstract]) OR (“Cameroon"[Title/Abstract]) OR (“Chad"[Title/Abstract]) OR (“Comoros"[Title/Abstract]) OR (“Congo"[Title/Abstract]) OR (“Cote d'Ivoire"[Title/Abstract]) OR (“Ivory Coast"[Title/Abstract]) OR (“Djibouti"[Title/Abstract]) OR (“Eritrea"[Title/Abstract]) OR (“Eswatini"[Title/Abstract]) OR (“Swaziland"[Title/Abstract]) OR (“Ethiopia"[Title/Abstract]) OR (“Gabon"[Title/Abstract]) OR (“Gambia"[Title/Abstract]) OR (“Ghana"[Title/Abstract]) OR (“Guinea*"[Title/Abstract]) OR (“Kenya"[Title/Abstract]) OR (“Lesotho"[Title/Abstract]) OR (“Liberia"[Title/Abstract]) OR (“Madagascar"[Title/Abstract]) OR (“Malawi"[Title/Abstract]) OR (“Mali"[Title/Abstract]) OR (“Mauritania"[Title/Abstract]) OR (“Mauritius"[Title/Abstract]) OR (“Mozambique"[Title/Abstract]) OR (“Namibia"[Title/Abstract]) OR (“Niger*"[Title/Abstract]) OR (“Rwanda"[Title/Abstract]) OR (“Sao Tome and Principe"[Title/Abstract]) OR (“Senegal"[Title/Abstract]) OR (“Seychelles"[Title/Abstract]) OR (“Sierra Leone"[Title/Abstract]) OR (“Somalia"[Title/Abstract]) OR (“Sudan"[Title/Abstract]) OR (“Tanzania"[Title/Abstract]) OR (“Togo"[Title/Abstract]) OR (“Uganda"[Title/Abstract]) OR (“Zambia"[Title/Abstract]) OR (“Zimbabwe"[Title/Abstract])) |
|  | AND |
| Date | 1^st^ January 2012 to present |
