# Supplementary material for: Antimicrobial resistance in bacterial wound, skin, soft tissue and surgical site infections in Central, Eastern, Southern and Western Africa: A systematic review and meta-analysis
Source: PLOS Glob Public Health. 2024 Apr 16;4(4):e0003077. doi: 10.1371/journal.pgph.0003077 (PMC11020607; doi:10.1371/journal.pgph.0003077)
Supplement: S2 Text — (DOCX) [file pgph.0003077.s003.docx]

**S2 Text: Antibiotic agents and classes of interest according to species**

*Staphylococcus aureus*:

- Aminoglycosides (gentamicin)
- Ansamycins (rifampicin or rifampin)
- Anti-MRSA cephalosporins (ceftaroline)
- Anti-staphylococcal beta-lactams or cephamycins (cefoxitin, methicillin or oxacillin)
- Fluoroquinolones (ciprofloxacin or moxifloxacin)
- Folate synthase inhibitors (trimethoprim-sulphamethoxazole)
- Fucidanes (fusidic acid)
- Glycopeptides (teicoplanin, telavancin or vancomycin)
- Glycyclines (tigecycline)
- Lincosamides (clindamycin)
- Lipopeptides (daptomycin)
- Macrolides (erythromycin)
- Oxazolidinones (linezolid)
- Phenicols (chloramphenicol)
- Phosphonic acids (fosfomycin)
- Streptogramins (quinopristin-dalfopristin)
- Tetracyclines (doxycycline, minocycline or tetracycline)

*Escherichia coli* and *Klebsiella pneumoniae*:

- Aminoglycosides (amikacin, gentamicin, netilmicin or tobramycin)
- Anti-MRSA cephalosporins (ceftaroline)
- Anti-pseudomonal penicillins with beta-lactamases (piperacillin-tazobactam or ticaracillin-clavalanic acid)
- Carbapenems (doripenem, etrapenem, imipenem or meropenem)
- Non-extended spectrum (first and second generation) cephalosporins (cefazolin or cefuroxime)
- Extended spectrum (third and fourth generation) cephalosporins (cefepime, cefotaxime, ceftazidime or ceftriaxone)
- Caphamycins (cefotetan or cefoxitin)
- Fluoroquinolones (ciprofloxacin)
- Folate synthesis inhibitors (trimethoprim-sulphamethoxazole)
- Glycylcyclines (tigecycline)
- Monobactams (aztreonam)
- Penicillins (ampicillin)
- Penicillins with beta-lactamase inhibitors (amoxicillin-clavulanic acid or ampicillin-salbactam)
- Phenicols (chloramphenicol)
- Phosphonic acids (fosfomycin)
- Polymyxins (colistin)
- Tetracyclines (doxycycline, minocycline or tetracycline)

*Pseudomonas aeruginosa*:

- Aminoglycosides (amikacin, gentamicin, netilmicin or tobramycin)
- Anti-pseudomonal carbapenems (doripenem, imipenem or meropenem)
- Anti-pseudomonal cephalosporins (cefepime or ceftazidime)
- Anti-pseudomonal fluoroquinolones (ciprofloxacin or levofloxacin)
- Anti-pseudomonal penicillins with beta-lactamases (piperacillin-tazobactam or ticaracillin-clavalanic acid)
- Phosphonic acids (fosfomycin)
- Monobactams (aztreonam)
- Polymyxins (colistin or polymyxin B)

*Acinetobacter baumannii*:

- Aminoglycosides (amikacin, gentamicin, netilmicin or tobramycin)
- Anti-pseudomonal carbapenems (doripenem, imipenem or meropenem)
- Anti-pseudomonal fluoroquinolones (ciprofloxacin or levofloxacin)
- Anti-pseudomonal penicillins with beta-lactamases (piperacillin-tazobactam or ticaracillin-clavalanic acid)
- Extended spectrum (third and fourth generation) cephalosporins (cefepime, cefotaxime, ceftazidime or ceftriaxone)
- Folate synthesis inhibitors (trimethoprim-sulphamethoxazole)
- Penicillins with beta-lactamase inhibitors (ampicillin-salbactam)
- Polymyxins (colistin or polymyxin B)
- Tetracyclines (doxycycline, tetracycline or minocycline)
