# Supplementary material for: Antimicrobial resistance in bacterial wound, skin, soft tissue and surgical site infections in Central, Eastern, Southern and Western Africa: A systematic review and meta-analysis
Source: PLOS Glob Public Health. 2024 Apr 16;4(4):e0003077. doi: 10.1371/journal.pgph.0003077 (PMC11020607; doi:10.1371/journal.pgph.0003077)
Supplement: S3 Text — (DOCX) [file pgph.0003077.s004.docx]

**S3 Text: Study-level risk of bias assessment**

| 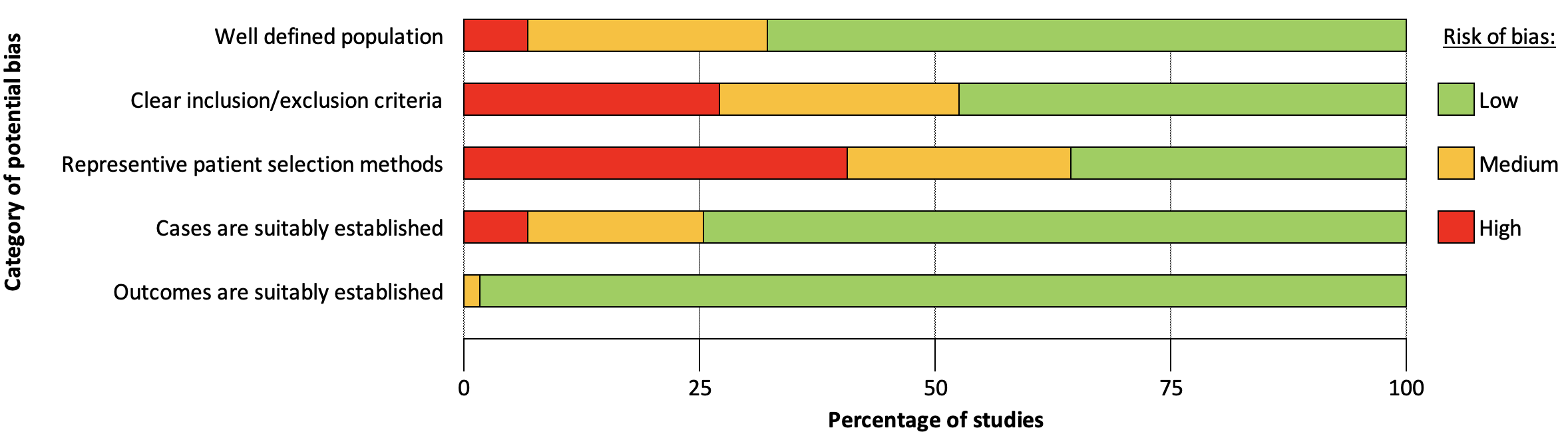  Study | Well defined population | Clear inclusion/exclusion criteria | Reprehensive patient selection methods | Cases suitably established | Outcomes suitably established | Study | Well defined population | Clear inclusion/exclusion criteria | Reprehensive patient selection methods | Cases suitably established | Outcomes suitably established |
| --- | --- | --- | --- | --- | --- | --- | --- | --- | --- | --- | --- |
| Abayneh 2022 |  |  |  |  |  | Mengesha 2014 |  |  |  |  |  |
| Abosse 2020 |  |  |  |  |  | Misha 2021 |  |  |  |  |  |
| Adeyemo 2021 |  |  |  |  |  | Moges 2019 |  |  |  |  |  |
| Akinloye 2021 |  |  |  |  |  | Mohammed 2013 |  |  |  |  |  |
| Alebel 2021 |  |  |  |  |  | Mohammed 2017 |  |  |  |  |  |
| Alelign 2022 |  |  |  |  |  | Monnheimer 2021 |  |  |  |  |  |
| Bediako-Bowan 2020 |  |  |  |  |  | Moremi 2019 |  |  |  |  |  |
| Bitew Kifilie 2018 |  |  |  |  |  | Motbainor 2020 |  |  |  |  |  |
| de Nardo 2016 |  |  |  |  |  | Muhindo 2021 |  |  |  |  |  |
| Desalegn 2020 |  |  |  |  |  | Mukagendaneza 2019 |  |  |  |  |  |
| Dessie 2016 |  |  |  |  |  | Nwankwo 2014 |  |  |  |  |  |
| Egyir 2021 |  |  |  |  |  | Oladeinde 2013 |  |  |  |  |  |
| Garoy 2019 |  |  |  |  |  | Omer 2020 |  |  |  |  |  |
| Garoy 2021 |  |  |  |  |  | Pondei 2013 |  |  |  |  |  |
| Gemechu 2021 |  |  |  |  |  | Rafai 2015 |  |  |  |  |  |
| George 2018 |  |  |  |  |  | Seni 2013 |  |  |  |  |  |
| Hope 2019 |  |  |  |  |  | Shakir 2021 |  |  |  |  |  |
| Janssen 2018 |  |  |  |  |  | Shimekaw 2020 |  |  |  |  |  |
| Kabanangi 2021 |  |  |  |  |  | Tadesse 2018 |  |  |  |  |  |
| Kahsay 2014 |  |  |  |  |  | Tambuwal 2020 |  |  |  |  |  |
| Kalayu 2019 |  |  |  |  |  | Tefera 2021 |  |  |  |  |  |
| Kassam 2017 |  |  |  |  |  | Tilahun 2022 (1) |  |  |  |  |  |
| Kazimoto 2018 |  |  |  |  |  | Tilahun 2022 (2) |  |  |  |  |  |
| Khalim 2021 |  |  |  |  |  | Tsige 2020 |  |  |  |  |  |
| Krumkamp 2020 |  |  |  |  |  | van der Meeren 2013 |  |  |  |  |  |
| Lakoh 2022 |  |  |  |  |  | Velin 2021 |  |  |  |  |  |
| Mama 2014 |  |  |  |  |  | Wangoye 2022 |  |  |  |  |  |
| Mama 2019 |  |  |  |  |  | Wekesa 2020 |  |  |  |  |  |
| Manyahi 2014 |  |  |  |  |  | Yagoup 2019 |  |  |  |  |  |
| Mekonnen 2021 |  |  |  |  |  |  |  |  |  |  |  |
