# Supplementary material for: Antimicrobial resistance in bacterial wound, skin, soft tissue and surgical site infections in Central, Eastern, Southern and Western Africa: A systematic review and meta-analysis
Source: PLOS Glob Public Health. 2024 Apr 16;4(4):e0003077. doi: 10.1371/journal.pgph.0003077 (PMC11020607; doi:10.1371/journal.pgph.0003077)
Supplement: S1 Table — (DOCX) [file pgph.0003077.s005.docx]

**S1 Table: Methods of bacterial species identification and susceptibility testing**

| Study | Culture agar | | | | | Species identification* | Sensitivity testing |
| --- | --- | --- | --- | --- | --- | --- | --- |
|  | BLD | CHO | MCY | MST | Other |  |  |
| Abayneh 2022 |  |  | + | + |  |  | Disk diffusion: CLSI |
| Abosse 2020 | + |  | + | + |  |  | Disk diffusion: CLSI |
| Adeyemo 2021 | + | + | + |  |  |  | Disk diffusion: CLSI (modified) |
| Akinloye 2021 | + |  | + |  |  |  | Disk diffusion: CLSI |
| Alebel 2021 | + |  | + |  |  | All isolates confirmed by VITEK 2 Compact | Disk diffusion: CLSI |
| Alelign 2022 | + |  | + | + |  |  | Disk diffusion: CLSI |
| Bediako-Bowan 2020 | + | + | + |  |  | MALDI-TOF Biotyper only | Disk diffusion: EUCAST |
| Bitew Kifilie 2018 | + |  | + | + |  |  | Disk diffusion: CLSI |
| De Nardo 2016 | + |  | + |  |  |  | Disk diffusion: EUCAST |
| Desalegn 2020 | + |  |  | + |  |  | Disk diffusion: CLSI (modified) |
| Dessie 2016 | + |  | + | + |  |  | Disk diffusion: CLSI |
| Egyir 2021 | + |  |  | + |  | All isolates confirmed by MALDI-TOF-MS | Disk diffusion: CLSI |
| Garoy 2019 |  |  |  | + | Nutrient |  | Disk diffusion: CLSI |
| Garoy 2021 |  | + | + | + |  |  | Disk diffusion: CLSI |
| Gemechu 2021 | + |  | + |  |  |  | Disk diffusion: CLSI |
| George 2018 | + | + | + |  |  |  | Disk diffusion: CLSI |
| Hope 2019 | + | + | + |  |  | API 20E if inadequate identification | Disk diffusion: CLSI |
| Janssen 2018 | + |  | + |  |  | All isolates confirmed by MALDI Biotyper 3.0 | VITEK 2 |
| Kabanangi 2021 | + |  | + |  |  |  | Disk diffusion: CLSI |
| Kahsay 2014 | + |  |  | + |  |  | Disk diffusion: CLSI |
| Kalayu 2019 | + |  | + | + |  |  | Disk diffusion: CLSI |
| Kassam 2017 | + |  | + |  |  |  | Disk diffusion: CLSI |
| Kazimoto 2018 | + | + | + |  |  | Methods included API | Disk diffusion: CLSI, MDR confirmed by VITEK2 |
| Khalim 2019 | + | + | + | + | XLD |  | Broth microdilution: CLSI |
| Krumkamp 2020 | + |  | + |  | C-CNA | MALDI Biotyper 3.0 only | Disk diffusion: CLSI, confirmed by VITEK 2 |
| Lakoh 2022 |  |  |  |  | CO | Methods included VITEK 2 Compact | VITEK 2 Compact |
| Mama 2014 | + |  | + |  |  |  | Disk diffusion: CLSI |
| Mama 2019 |  |  |  | + |  |  | Disk diffusion: CLSI |
| Manyahi 2014 | + |  | + |  |  | Methods included VITEK and API 20E | Disk diffusion: CLSI |
| Mekonnen 2021 | + |  | + |  |  |  | Disk diffusion: CLSI |
| Mengesha 2014 | + |  | + | + |  |  | Disk diffusion: CLSI |
| Misha 2021 | + | + | + |  |  |  | Disk diffusion: CLSI |
| Moges 2019 | As per local guidelines | | | |  | All isolates confirmed by VITEK 2 Compact | Disk diffusion: CLSI |
| Mohammed 2013 | + | + | + |  |  |  | Disk diffusion: CLSI (modified) |
| Mohammed 2017 |  |  | + | + | PAB |  | Disk diffusion: CLSI |
| Monnheimer 2021 | + |  | + |  |  | MALDI Biotyper 3.0 only | VITEK 2 |
| Moremi 2019 |  |  |  |  | CBA | All isolates confirmed by VITEK MS | VITEK 2 |
| Motbainor 2020 | + |  | + |  |  | All isolates confirmed by VITEK 2 Compact | Disk diffusion: CLSI |
| Muhindo 2021 |  | + | + |  | Nutrient | Methods included API 20E/staph | Disk diffusion: CLSI |
| Mukagendaneza 2019 | + |  | + |  |  |  | Disk diffusion: CLSI |
| Nwankwo 2014 | + |  | + | + | Sabourand |  | Disk diffusion: CLSI |
| Oladeinde 2013 | + |  | + |  | Sabourand |  | Disk diffusion: BSAC |
| Omer 2020 |  |  | + |  | Cetrimide |  | Disk diffusion: CLSI (modified) |
| Pondei 2013 | + | + | + |  | CLED |  | Disk diffusion: NCCLS |
| Rafai 2015 | + | + |  |  | BCP |  | Disk diffusion: SFM |
| Seni 2013 | + |  |  | + |  |  | Disk diffusion: CLSI |
| Shakir 2021 | + |  | + | + |  |  | Disk diffusion: CLSI |
| Shimekaw 2020 | + |  | + | + |  |  | Disk diffusion: CLSI (modified) |
| Tadesse 2018 |  |  |  | + |  |  | Disk diffusion: CLSI |
| Tambuwal 2020 | + |  |  | + |  |  | Disk diffusion: CLSI |
| Tefera 2021 | + |  |  | + |  |  | Disk diffusion: CLSI (modified) |
| Tilahun 2022 (1) | + |  | + |  | Nutrient | VITEK 2 Compact if inadequate identification | Disk diffusion: CLSI |
| Tilahun 2022 (2) | + | + | + | + |  |  | Disk diffusion: CLSI |
| Tsige 2020 | + |  |  | + |  |  | Disk diffusion: CLSI |
| Van der Meeren 2013 | + |  | + | + |  |  | Disk diffusion: CLSI |
| Velin 2021 | + |  |  |  |  | Methods included VIKEK 2 | VITEK 2 |
| Wangoye 2022 | + | + | + |  |  |  | Broth microdilution: CLSI |
| Wekesa 2020 | + | + | + |  |  |  | Disk diffusion: CLSI |
| Yagoup 2019 | + |  | + |  | Cetrimide |  | Disk diffusion: CLSI |
| *Included standard morphology, Gram stain and biochemical tests according to local policy in all studies, unless specified otherwise. API = analytical profile index, BCP = bromocresol purple lactose, BLD = blood, BSAC = British Society for Antimicrobial Chemotherapy, C-CNA = Columbia colistin nalidixic acid, CHO = chocolate, CLED = cystine lactose electrolyte deficient, CLSI = Clinical and Laboratory Standards Institute, CO = CHROMagar Orientation, EUCAST = European Committee on Antimicrobial Susceptibility Testing, MALDI-TOF = matrix-assisted laser desorption ionisation time-of-flight, MCY = MacConkey, MDR = multidrug resistance, MST = mannitol salt, NCCLS = National Committee for Clinical Laboratory Standards, PAB = *Pseudomonas* agar base, SFM = Société Française de Microbiologie, XLD = xylose lysine deoxycholate. | | | | | | | |
