# Supplementary material for: Antimicrobial resistance in bacterial wound, skin, soft tissue and surgical site infections in Central, Eastern, Southern and Western Africa: A systematic review and meta-analysis
Source: PLOS Glob Public Health. 2024 Apr 16;4(4):e0003077. doi: 10.1371/journal.pgph.0003077 (PMC11020607; doi:10.1371/journal.pgph.0003077)
Supplement: S2 Table — (DOCX) [file pgph.0003077.s006.docx]

| **S2 Table: Regional AMR estimates** | **Central Africa** | | | **Eastern Africa** | | | **Southern Africa** | | | **Western Africa** | | |
| --- | --- | --- | --- | --- | --- | --- | --- | --- | --- | --- | --- | --- |
| Antibiotic class (agents) | Resistance (95% CI) | Samples (studies) | I^2^ (%) | Resistance (95% CI) | Samples (studies) | I^2^ (%) | Resistance (95% CI) | Samples (studies) | I^2^ (%) | Resistance (95% CI) | Samples (studies) | I^2^ (%) |
| ***Staphylococcus aureus*** | | | | | | | | | | | | |
| Aminoglycosides  (Gentamycin) | 0.19 (0.13 - 0.27) | 127 (1) | - | 0.28 (0.15 - 0.42) | 885 (20) | 95 | 0.11 (0.06 - 0.19) | 99 (1) | - | 0.15  (0.02 - 0.34) | 311 (7) | 91 |
| Ansamycins  (Rifampin) | - | - | - | 0.07 (0.03 - 0.12) | 152 (2) | - | - | - | - | 0.00  (0.00 - 0.11) | 31 (1) | - |
| Anti-staphylococcal beta-lactams/cephamycins  (Cefoxitin, methicillin or oxacillin) | 0.48  (0.40 - 0.55) | 171 (2) | - | 0.58 (0.41 - 0.73) | 1094 (23) | 96 | 0.10 (0.06 - 0.18) | 99 (1) | - | 0.23 (0.09 - 0.41) | 247 (6) | 88 |
| Fluoroquinolones  (Ciprofloxacin) | 0.10 (0.06 - 0.17) | 127 (1) | - | 0.20 (0.12 - 0.29) | 812 (16) | 88 | 0.12 (0.07 - 0.20) | 99 (1) | - | 0.22 (0.10 - 0.37) | 447 (5) | 89 |
| Folate synthesis inhibitors  (Cotrimoxazole) | 0.39 (0.29 - 0.50) | 129 (2) | - | 0.57 (0.40 - 0.73) | 906 (19) | 96 | 0.19 (0.13 - 0.28) | 99 (1) | - | 0.42 (0.00 - 0.94) | 298 (5) | 99 |
| Glycopeptides (Vancomycin) | 0.00 (0.00 - 0.66) | 2 (1) | - | 0.06 (0.00 - 0.15) | 584 (12) | 92 | 0.00 (0.00 - 0.04) | 99 (1) | - | 0.00 (0.00 - 0.03) | 65 (2) | - |
| Lincosamides  (Clindamycin) | 0.09 (0.05 - 0.16) | 127 (1) | - | 0.32 (0.17 - 0.49) | 828 (16) | 96 | 0.02 (0.01 - 0.07) | 99 (1) | - | 0.06 (0.01 - 0.13) | 105 (4) | 26 |
| Macrolides  (Erythromycin) | 0.54 (0.46 - 0.63) | 127 (1) | - | 0.52 (0.37 - 0.67) | 829 (20) | 94 | 0.21 (0.14 - 0.30) | 99 (1) | - | 0.21 (0.07 - 0.39) | 298 (5) | 87 |
| Oxazolidinones  (Linezolid) | No studies | No studies | - | No studies | No studies | - | No studies | No studies | - | 0.00 (0.00 - 0.10) | 33 (1) | - |
| Phenicols  (Chloramphenicol) | No studies | No studies | - | 0.33 (0.17 - 0.50) | 762 (15) | 95 | 0.09 (0.05 - 0.16) | 99 (1) | - | 1.00 (0.98 - 1.00) | 162 (1) | - |
| Phosphoric acids  (Fosfomycin) | No studies | No studies | - | No studies | No studies | - | No studies | No studies | - | 0.00 (0.00 - 0.11) | 31 (1) | - |
| Tetracyclines  (Doxycycline or tetracycline) | 0.50 (0.39 - 0.60) | 129 (2) | - | 0.50 (0.37 - 0.63) | 897 (18) | 93 | 0.58 (0.48 - 0.67) | 99 (1) | - | 0.68 (0.28 - 0.97) | 267 (5) | 97 |
| ***Escherichia coli*** | | | | | | | | | | | | |
| Aminoglycosides  (Amikacin or gentamicin) | 0.33 (0.06 - 0.79) | 3 (1) | - | 0.49 (0.39 - 0.60) | 389 (18) | 72 | No studies | No studies | - | 0.61 (0.38 - 0.82) | 271 (4) | 86 |
| Anti-pseudomonal penicillins with beta-lactamase inhibitors (Piperacillin-tazobactam) | No studies | No studies | - | 0.42 (0.32 - 0.52) | 91 (2) | - | No studies | No studies | - | 0.31 (0.24 - 0.39) | 158 (2) | - |
| Carbapenems (Imipenem or meropenem) | 0.00 (0.00 - 0.56) | 3 (1) | - | 0.10 (0.00 - 0.28) | 257 (10) | 90 | No studies | No studies | - | 0.00 (0.00 - 0.02) | 158 (2) | - |
| First and second generation cephalosporins  (Cefazolin or cefuroxime) | No studies | No studies | - | 0.82 (0.65 - 0.94) | 55 (4) | 37 | No studies | No studies | - | 0.62 (0.54 - 0.69) | 158 (2) | - |
| Third and fourth generation cephalosporins  (Cefepime, cefotaxime, ceftazidime or ceftriaxone) | 1.00 (0.44 - 1.00) | 3 (1) | - | 0.76 (0.66 - 0.85) | 388 (19) | 72 | No studies | No studies | - | 0.56 (0.22 - 0.87) | 336 (5) | 95 |
| Cephamycins (Cefoxitin) | No studies | No studies | - | 0.52 (0.32 - 0.71) | 131 (5) | 71 | No studies | No studies | - | No studies | No studies | - |
| Fluoroquinolones (Ciprofloxacin) | 0.33 (0.06 - 0.79) | 3 (1) | - | 0.51 (0.40 - 0.62) | 378 (19) | 72 | No studies | No studies | - | 0.64 (0.26 - 0.95) | 450 (4) | 98 |
| Folate pathway inhibitors (Cotrimoxazole) | 1.00 (0.44 - 1.00) | 3 (1) | - | 0.79 (0.68 - 0.89) | 351 (17) | 77 | No studies | No studies | - | 0.96 (0.94 - 0.98) | 378 (2) | - |
| Penicillins (Ampicillin) | 1.00 (0.44 - 1.00) | 3 (1) | - | 0.92 (0.83 - 0.99) | 364 (18) | 79 | No studies | No studies | - | 0.94 (0.91 - 0.96) | 397 (3) | - |
| Penicillins with beta-lactamase inhibitors (Amoxicillin-clavulanic acid) | 0.00 (0.00 - 0.56) | 3 (1) | - | 0.89 (0.83 - 0.94) | 288 (13) | 34 | No studies | No studies | - | 0.59 (0.35 - 0.82) | 438 (4) | 95 |
| Phenicols (Chloramphenicol) | No studies | No studies | - | 0.45 (0.34 - 0.56) | 333 (14) | 70 | No studies | No studies | - | 1.00 (0.93 - 1.00) | 54 (1) | - |
| Tetracyclines  (Doxycycline or tetracycline) | 0.67 (0.21 - 0.94) | 3 (1) | - | 0.79 (0.69 - 0.87) | 346 (15) | 98 | No studies | No studies | - | 1.00 (0.93 - 1.00) | 54 (1) | - |
| ***Klebsiella pneumoniae*** | | | | | | | | | | | | |
| Aminoglycosides  (Amikacin or gentamicin) | 1.00 (0.68 - 1.00) | 8 (1) | - | 0.29 (0.09 - 0.52) | 124 (8) | 85 | No studies | No studies | - | 0.42 (0.29 - 0.55) | 69 (3) | - |
| Anti-pseudomonal penicillins with beta-lactamase inhibitors (Piperacillin-tazobactam) | No studies | No studies | - | 0.78 (0.45 - 0.94) | 9 (1) | 1 | No studies | No studies | - | 0.54 (0.40 - 0.69) | 48 (2) | - |
| Carbapenems (Imipenem or meropenem) | 0.00 (0.00 - 0.32) | 8 (1) | - | 0.31 (0.05 - 0.64) | 68 (4) | 85 | No studies | No studies | - | 0.00 (0.00 - 0.00) | 186 (4) | 89 |
| First and second generation cephalosporins  (Cefazolin or cefuroxime) | No studies | No studies | - | 0.25 (0.11 - 0.42) | 33 (2) | - | No studies | No studies | - | 0.80 (0.43 - 1.00) | 69 (3) | - |
| Third and fourth generation cephalosporins  (Cefepime, cefotaxime, ceftazidime or ceftriaxone) | 1.00 (0.68 - 1.00) | 8 (1) | - | 0.65 (0.36 - 0.90) | 131 (9) | 89 | No studies | No studies | - | 0.43 (0.18 - 0.69) | 117 (4) | 87 |
| Cephamycins (Cefoxitin) | No studies | No studies | - | 0.43 (0.10 - 0.79) | 57 (3) | - | No studies | No studies | - | No studies | No studies | - |
| Fluoroquinolones (Ciprofloxacin) | 0.25 (0.07 - 0.59) | 8 (1) | - | 0.26 (0.07 - 0.52) | 129 (8) | 88 | No studies | No studies | - | 0.45 (0.16 - 0.76) | 117 (4) | 91 |
| Folate pathway inhibitors (Cotrimoxazole) | 0.75 (0.41 - 0.93) | 8 (1) | - | 0.76 (0.58 - 0.91) | 136 (9) | 78 | No studies | No studies | - | 0.81 (0.48 - 1.00) | 117 (4) | 92 |
| Penicillins with beta-lactamase inhibitors (Amoxicillin-clavulanic acid) | 1.00 (0.68 - 1.00) | 8 (1) | - | 0.88 (0.71 - 0.99) | 89 (6) | 70 | No studies | No studies | - | 0.62 (0.51 - 0.72) | 83 (2) | - |
| Phenicols (Chloramphenicol) | No studies | No studies | - | 0.51 (0.28 - 0.74) | 99 (6) | 80 | No studies | No studies | - | No studies | No studies | - |
| Tetracyclines  (Doxycycline or tetracycline) | 0.88 (0.53 - 0.98) | 8 (1) | - | 0.67 (0.47 - 0.85) | 135 (9) | 80 | No studies | No studies | - | No studies | No studies | - |
| ***Pseudomonas aeruginosa*** | | | | | | | | | | | | |
| Aminoglycosides  (Amikacin or gentamicin) | 0.00 (0.00 - 0.66) | 2 (1) | - | 0.19 (0.06 - 0.35) | 287 (18) | 3 | No studies | No studies | - | 0.23 (0.06 - 0.46) | 301 (6) | 94 |
| Anti-pseudomonal carbapenems (Imipenem or meropenem) | 0.00 (0.00 - 0.16) | 10 (2) | - | 0.19 (0.02 - 0.43) | 256 (10) | 94 | No studies | No studies | - | 0.45 (0.00 - 1.00) | 138 (3) | - |
| Anti-pseudomonal cephalosporins  (Cefepime or ceftazidime) | 0.00 (0.00 - 0.66) | 2 (1) | - | 0.45 (0.23 - 0.67) | 222 (12) | 87 | No studies | No studies | - | 0.38 (0.10 - 0.72) | 376 (7) | 98 |
| Antipseudomonal fluoroquinolones (Ciprofloxacin) | 0.00 (0.00 - 0.66) | 2 (1) | - | 0.20 (0.06 - 0.38) | 331 (18) | 89 | No studies | No studies | - | 0.45 (0.20 - 0.71) | 337 (6) | 96 |
| Anti-pseudomonal penicillins with beta-lactamase inhibitors (Piperacillin-tazobactam) | No studies | No studies | - | 0.45 (0.29 - 0.62) | 37 (2) | - | No studies | No studies | - | 0.10 (0.03 - 0.30) | 20 (1) | - |
| Monobactams (Aztreonam) | No studies | No studies | - | 0.79 (0.67 - 88) | 53 (1) | - | No studies | No studies | - | 0.93 (0.83 - 0.97) | 69 (1) | - |
| Polymyxins (Polymyxin B) | No studies | No studies | - | No studies | No studies | - | No studies | No studies | - | 0.25 (0.16 - 0.36) | 69 (1) | - |
| ***Acinetobacter baumannii*** | | | | | | | | | | | | |
| Aminoglycosides  (Gentamicin) | 0.56 (0.27 - 0.81) | 9 (1) | - | 0.50 (0.08 - 0.92) | 30 (3) | - | No studies | No studies | - | 0.61 (0.43 - 0.79) | 31 (2) | - |
| Anti-pseudomonal carbapenems (Imipenem or meropenem) | 0.03 (0.00 - 0.18) | 20 (2) | - | 0.37 (0.19 - 0.56) | 30 (3) | - | No studies | No studies | - | 0.22 (0.08 - 0.39) | 31 (2) | - |
| Antipseudomonal fluoroquinolones (Ciprofloxacin) | 0.44 (0.19 - 0.73) | 9 (1) | - | 0.47 (0.28 - 0.66) | 30 (3) | - | No studies | No studies | - | 0.38 (0.14 - 0.69) | 8 (1) | - |
| Anti-pseudomonal penicillins with beta-lactamase inhibitors (Piperacillin-tazobactam) | No studies | No studies | - | 0.50 (0.19 - 0.81) | 6 (1) | - | No studies | No studies | - | 0.00 (0.00 - 0.14) | 23 (1) | - |
| Extended-spectrum cephalosporins (Cefepime, cefotaxime, ceftazidime or ceftriaxone) | 1.00 (0.70 - 1.00) | 9 (1) | - | 0.94 (0.64 - 1.00) | 31 (4) | 53 | No studies | No studies | - | 0.03 (0.00 - 0.15) | 31 (2) | - |
| Folate pathway inhibitors (Cotrimoxazole) | 0.67 (0.35 - 0.88) | 9 (1) | - | 0.67 (0.30 - 0.90) | 6 (1) | - | No studies | No studies | - | No studies | No studies | - |
