# Supplementary material for: Antimicrobial resistance in bacterial wound, skin, soft tissue and surgical site infections in Central, Eastern, Southern and Western Africa: A systematic review and meta-analysis
Source: PLOS Glob Public Health. 2024 Apr 16;4(4):e0003077. doi: 10.1371/journal.pgph.0003077 (PMC11020607; doi:10.1371/journal.pgph.0003077)
Supplement: S3 Table — (DOCX) [file pgph.0003077.s007.docx]

**S3 Table: SSI Pprospective cohort study versus cross-sectional study sensitivity analysis**

|  | **Prospective cohort studies** | | | **Cross-sectional studies** | | |
| --- | --- | --- | --- | --- | --- | --- |
|  | Resistance (95% CI) | Samples (studies) | I^2^ (%) | Resistance (95% CI) | Samples (studies) | I^2^ (%) |
| ***Staphylococcus aureus*** | | | | | | |
| Aminoglycosides  (Gentamicin) | 0.05 (0.00 - 0.17) | 71 (3) | - | 0.42 (0.21 - 0.64) | 388 (12) | 94 |
| Ansamycins  (Rifampin) | No studies | No studies | - | 0.35 (0.18 - 0.57) | 20 (1) | - |
| Anti-staphylococcal beta-lactams/cephamycins  (Cefoxitin, methicillin or oxacillin) | 0.28 (0.06 - 0.57) | 142 (6) | 91 | 0.59 (0.41 - 0.77) | 642 (16) | 95 |
| Fluoroquinolones  (Ciprofloxacin) | 0.21 (0.05 - 0.43) | 87 (4) | 77 | 0.37 (0.29 - 0.44) | 487 (7) | 55 |
| Folate synthesis inhibitors  (Cotrimoxazole) | 0.42 (0.18 - 0.68) | 94 (6) | 77 | 0.78 (0.58 - 0.93) | 612 (11) | 96 |
| Glycopeptides (Vancomycin) | 0.00 (0.00 - 0.20) | 34 (3) | - | 0.12 (0.01 - 0.29) | 334 (7) | 93 |
| Lincosamides  (Clindamycin) | 0.35 (0.05 - 0.72) | 73 (4) | 87 | 0.38 (0.20 - 0.58) | 446 (10) | 94 |
| Macrolides  (Erythromycin) | 0.36 (0.17 - 0.58) | 92 (5) | 71 | 0.61 (0.45 - 0.77) | 706 (14) | 94 |
| Oxazolidinones  (Linezolid) | 0.00 (0.00 - 0.10) | 33 (1) | - | No studies | No studies | - |
| Phenicols  (Chloramphenicol) | 0.32 (0.08 - 0.62) | 13 (2) | - | 0.51 (0.25 - 0.77) | 308 (7) | 95 |
| Phosphoric acids  (Fosfomycin) | No studies | No studies | - | No studies | No studies | - |
| Tetracyclines  (Doxycycline or tetracycline) | 0.34 (0.11 - 0.60) | 67 (5) | 65 | 0.54 (0.39 - 0.70) | 437 (10) | 90 |
| ***Escherichia coli*** | | | | | | |
| Aminoglycosides  (Amikacin or gentamicin) | 0.35 (0.17 - 0.55) | 198 (6) | 74 | 0.58 (0.47 - 0.69) | 256 (10) | 58 |
| Anti-pseudomonal penicillins with beta-lactamase inhibitors (Piperacillin-tazobactam) | 0.28 (0.21 - 0.36) | 139 (1) | - | 0.31 (0.21 - 0.42) | 72 (1) | - |
| Carbapenems (Imipenem or meropenem) | 0.00 (0.00 - 0.05) | 174 (5) | 32 | 0.13 (0.00 - 0.41) | 206 (6) | 94 |
| First and second generation cephalosporins  (Cefazolin or cefuroxime) | 0.74 (0.54 - 0.91) | 183 (4) | 74 | 0.82 (0.52 - 0.95) | 11 (1) | - |
| Third and fourth generation cephalosporins  (Cefepime, cefotaxime, ceftazidime or ceftriaxone) | 0.82 (0.69 - 0.92) | 59 (5) | 0 | 0.78 0.56 - 0.94) | 501 (12) | 95 |
| Cephamycins (Cefoxitin) | 0.82 (0.55 - 0.95) | 12 (1) | - | 0.44 (0.27 - 0.62) | 119 (4) | 57 |
| Fluoroquinolones (Ciprofloxacin) | 0.67 (0.39 - 0.90) | 198 (6) | 86 | 0.57 (0.44 - 0.69) | 477 (11) | 76 |
| Folate pathway inhibitors (Cotrimoxazole) | 0.85 (0.55 - 1.00) | 174 (5) | 85 | 0.88 (0.78 - 0.96) | 488 (11) | 82 |
| Penicillins (Ampicillin) | 0.96 (0.84 - 1.00) | 198 (6) | 65 | 0.96 (0.90 - 1.00) | 484 (12) | 65 |
| Penicillins with beta-lactamase inhibitors (Amoxicillin-clavulanic acid) | 0.76 (0.57 - 0.92) | 189 (5) | 71 | 0.80 (0.54 - 0.98) | 440 (8) | 95 |
| Phenicols (Chloramphicenol) | 0.47 (0.15 - 0.81) | 56 (4) | 84 | 0.44 (0.33 - 0.56) | 214 (7) | 50 |
| Tetracyclines  (Doxycycline or tetracycline) | (0.62 - 0.92) | 39 (3) | - | 0.84 (0.73 - 0.93) | 247 (10) | 66 |
| ***Klebsiella pneumoniae*** | | | | | | |
| Aminoglycosides  (Amikacin or gentamicin) | 0.66 (0.27 - 0.97) | 53 (3) | - | 0.43 (0.23 - 0.64) | 57 (3) | - |
| Anti-pseudomonal penicillins with beta-lactamase inhibitors (Piperacillin-tazobactam) | 0.57 (0.41 - 0.72) | 35 (1) | - | No studies | No studies | - |
| Carbapenems (Imipenem or meropenem) | 0.00 (0.00 - 0.03) | 43 (2) | - | 0.24 (0.00 - 0.64) | 59 (3) | - |
| First and second generation cephalosporins  (Cefazolin or cefuroxime) | 0.79 (0.65 - 0.90) | 45 (2) | - | No studies | No studies | - |
| Third and fourth generation cephalosporins  (Cefepime, cefotaxime, ceftazidime or ceftriaxone) | 0.88 (0.68 - 0.99) | 53 (3) | - | 0.66 (0.4 - 0.88) | 123 (6) | 83 |
| Cephamycins (Cefoxitin) | No studies | No studies | - | 0.43 (0.10 - 0.79) | 57 (3) | - |
| Fluoroquinolones (Ciprofloxacin) | 0.45 (0.05 - 0.90) | 53 (3) | - | 0.36 (0.10 - 0.66) | 121 (5) | 90 |
| Folate pathway inhibitors (Cotrimoxazole) | 0.82 (0.69 - 0.93) | 43 (2) |  | 0.88 (0.69 - 0.99) | 127 (6) | 82 |
| Penicillins with beta-lactamase inhibitors (Amoxicillin-clavulanic acid) | 0.94 (0.76 - 1.00) | 53 (3) | - | 0.69 (0.44 - 0.89) | 107 (4) | 83 |
| Phenicols (Chloramphenicol) | 0.40 (0.17 - 0.69) | 10 (1) | - | 0.56 (0.40 - 0.71) | 43 (2) | - |
| Tetracyclines  (Doxycycline or tetracycline) | 0.57 (0.32 - 0.80) | 18 (2) | - | 0.82 (0.65 - 0.94) | 79 (5) | 57 |
| ***Pseudomonas aeruginosa*** | | | | | | |
| Aminoglycosides  (Amikacin or gentamicin) | 0.03 (0.00 - 0.23) | 120 (5) | 79 | 0.31 (0.10 - 0.54) | 96 (9) | 66 |
| Anti-pseudomonal carbapenems (Imipenem or meropenem) | 0.16 (0.00 - 0.59) | 114 (4) | 92 | 0.08 (0.00 - 0.24) | 89 (6) | 71 |
| Anti-pseudomonal cephalosporins  (Cefepime or ceftazidime) | 0.47 (0.04 - 0.92) | 65 (4) | 85 | 0.42 (0.23 - 0.62) | 150 (7) | 65 |
| Antipseudomonal fluoroquinolones (Ciprofloxacin) | 0.35 (0.01 - 0.90) | 120 (5) | 96 | 0.32 (0.07 - 0.62) | 162 (9) | 85 |
| Anti-pseudomonal penicillins with beta-lactamase inhibitors (Piperacillin-tazobactam) | No studies | No studies | - | 0.17 (0.05 - 0.45) | 12 (1) | - |
| Monobactams (Aztreonam) | No studies | No studies | - | No studies | No studies | - |
| Polymyxins (Polymyxin B) | No studies | No studies | - | No studies | No studies | - |
| ***Acinetobacter baumannii*** | | | | | | |
| Aminoglycosides  (Gentamicin) | 0.60 (0.41 - 0.77) | 32 (2) | - | 0.50 (0.08 - 0.92) | 30 (3) | - |
| Anti-pseudomonal carbapenems (Imipenem or meropenem) | 0.21 (0.08 - 0.38) | 32 (2) | - | 0.22 (0.03 - 0.50) | 41 (4) | 67 |
| Antipseudomonal fluoroquinolones (Ciprofloxacin) | 0.44 (0.19 - 0.73) | 9 (1) | - | 0.47 (0.28 - 0.66) | 30 (3) | - |
| Anti-pseudomonal penicillins with beta-lactamase inhibitors (Piperacillin-tazobactam) | 0.00 (0.00 - 0.14) | 23 (1) | - | 0.50 (0.19 - 0.81) | 6 (1) | - |
| Extended-spectrum cephalosporins (Cefepime, cefotaxime, ceftazidime or ceftriaxone) | 0.19 (0.06 - 0.36) | 32 (2) | - | 0.88 (0.56 - 1.00) | 30 (3) | - |
| Folate pathway inhibitors (Cotrimoxazole) | 0.67 (0.35 - 0.88) | 9 (1) | - | 0.67 (0.30 - 0.90) | 6 (1) | - |
| Tetracyclines  (Doxycycline or teracycline) | 0.07 (0.00 - 0.20) | 32 (1) | - | 0.74 (0.55 - 0.89) | 30 (3) | - |
